# A Report of a Rare Case of Rapidly Progressing Immature Teratoma With Associated Splenic Metastasis and Gliomatosis Peritonei

**DOI:** 10.7759/cureus.54080

**Published:** 2024-02-12

**Authors:** Anusha Adkoli, Colton Smith, Timothy Kennedy, James Aikins, Eugenia Girda

**Affiliations:** 1 Obstetrics and Gynecology, Rutgers Robert Wood Johnson Medical School, New Brunswick, USA; 2 Pathology and Laboratory Medicine, Rutgers Robert Wood Johnson Medical School, New Brunswick, USA; 3 Surgical Oncology, Rutgers Cancer Institute of New Jersey, New Brunswick, USA; 4 Gynecologic Oncology, Rutgers Cancer Institute of New Jersey, New Brunswick, USA

**Keywords:** splenic lesion, fertility sparing surgery, robotic-assisted surgery, gliomatosis peritonei, immature teratoma

## Abstract

Gliomatosis peritonei (GP) is a rare condition of mature glial tissue within the peritoneum often associated with immature teratomas. This was a case of rapid progression of immature teratoma with splenic lesions and associated GP. The patient was a 21-year-old female who presented with abdominal pain and CT imaging showing suspected malignant teratoma. The patient underwent exploratory laparotomy with fertility-sparing debulking surgery and was diagnosed with stage IIIC grade 3 immature teratoma. She then received adjuvant chemotherapy with bleomycin, etoposide, and cisplatin. Surveillance imaging demonstrated a non-avid splenic lesion. The tumor markers remained normal. She underwent robotic splenectomy and partial peritonectomy with intra-operative findings revealing numerous peritoneal nodules. Follow-up surveillance imaging showed no further lesions. The final histopathology examination demonstrated mature and mesenchymal neural tissue consistent with residual teratoma and no immature elements. The specimens were largely composed of nodules of mature glial tissue and focal areas of mature neuronal tissue. Immunohistochemistry demonstrated glial fibrillary acidic protein (GFAP) and S100 expression, confirming neural origin tissue. Octamer-binding transcription factor 4 (OCT-4) immunostain was negative which confirmed the absence of immature neural tissue. We report a rare case of rapid progression of immature teratoma with splenic metastasis and peritoneal nodules found ultimately to be mature teratoma and associated GP. Recognition of rapidly growing teratoma with new lesions as potential GP is imperative to prevent misdiagnosis as recurrence or progression of disease. This case was treated with secondary debulking surgery which should be a consideration of management if surgically feasible.

## Introduction

Gliomatosis peritonei (GP) is a rare condition of mature glial tissue within the peritoneum often associated with immature teratomas. Implants of benign glial tissue often involve the peritoneum, omentum, or lymph nodes [1]. Thus far, approximately 100 cases of GP have been reported [2]. Its origin is poorly understood and generally not associated with adverse outcomes. Given the limited number of cases that exist, there is no treatment algorithm that has been established. Cases have been reported of malignant transformation of glioma [3]. Other rare cases have been reported of mature teratomas with GP and increasing growth of metastatic disease [2]. Additionally, mature teratomas in splenic locations are rare with few cases reported [4]. This was a case of rapid progression of immature teratoma with splenic metastasis found to be mature teratoma with associated GP.

## Case presentation

**Figure 1:**
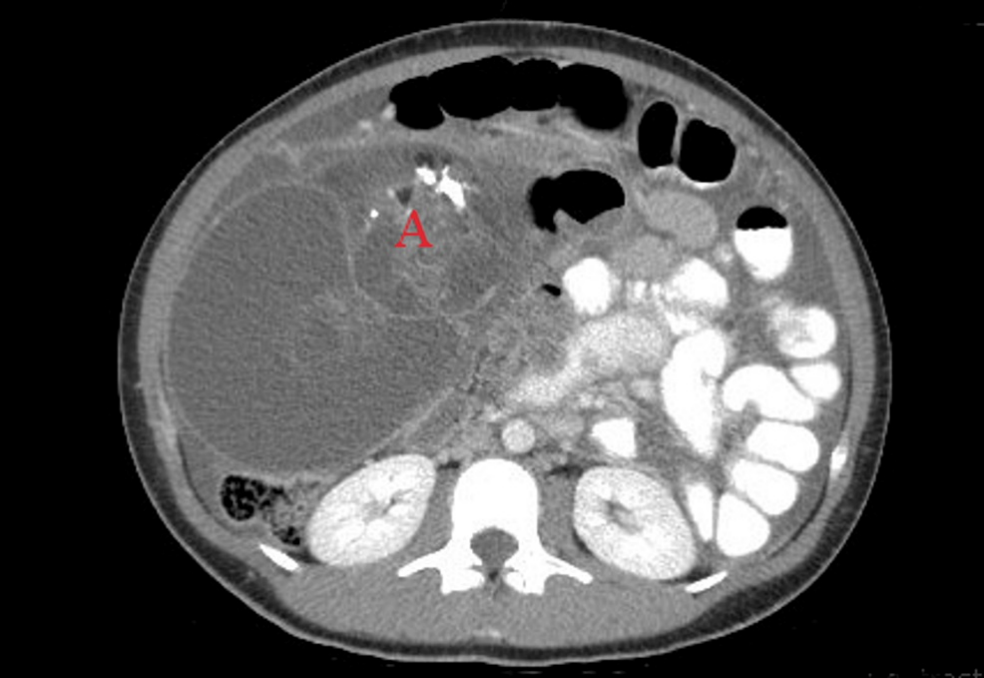
Representative of imaging prior to initial debulking surgery consistent with suspected malignant teratoma

The patient was a 21-year-old G0 female who presented with periumbilical abdominal pain. She had a few days of pain with mild abdominal cramping, bloating, and pain with palpation of the periumbilical region. She otherwise denied any other symptoms. On exam, a hard moderately distended abdomen was palpated with tenderness in the periumbilical region but without rebound or guarding. Computerized tomography (CT) imaging showed a large pelvic abdominal mass concerning ovarian neoplasm, likely immature ovarian teratoma, with omental caking, complex ascites, and multiple fat attenuating lesions suspected to be malignant teratoma. The mass measured approximately 27.4x15.6x17.2cm with multiple fatty elements and segmental irregular calcifications (Figure 1).

Work-up included tumor marker abnormalities which were significant for cancer antigen (CA) 125 = 129 (0.0-35), carcinoembryonic antigen (CEA) = 5.4 (0.0-3.0), and alpha-fetoprotein (AFP) = 64 (0.6-6). Lactate dehydrogenase (LDH) and beta-human chorionic gonadotropin (bhCG) levels were normal. The patient underwent exploratory laparotomy with fertility-sparing debulking surgery which included resection of pelvic mass, right salpingo-oophorectomy, omentectomy, right periaortic lymphadenectomy, bladder peritonectomy, resection of posterior cul-de-sac tumor, left adnexal tumor resection, removal of mesenteric nodules, organ ablation and cavitron ultrasonic surgical aspirator (CUSA) aspiration of multiple abdominal and pelvic peritoneal lesions.

Intra-operative findings included a 30-cm complex adnexal mass that was arising from the right adnexa and extending to the right upper quadrant, which had been preoperatively partially ruptured with sebaceous and fatty components in the ascites. A total of 3800 cc of serous ascitic fluid was removed from the abdomen. The left ovary contained several superficial nodules. The uterus appeared to be spared; however, uterine serosa, anterior bladder peritoneum, bilateral pelvic sidewalls, and posterior cul-de-sac were involved with several nodules. In addition, the posterior cul-de-sac had several complex cystic lesions measuring 2-5 cm that were removed from the rectovaginal septum, the left pelvic sidewall, and the right pelvic sidewall. The bladder peritoneum, perirectal mesentery, and right and left pericolic gutters contained tumor burden likely representing miliary disease which was controlled with argan ablation and CUSA. There were several nodules along the right diaphragm as well as a liver lesion that were resected. There was a lot of scarring noted between the anterior abdominal wall and the liver, but the diaphragmatic surfaces on the left appeared to be free of disease. The spleen was free of disease. The omentum was retracted and very fibrotic with several lymph nodes and omental lesions that measured about 1 cm, which were removed as part of the greater omentectomy. There was no bowel serosa involvement.

Several mesenteric lesions were removed via argan ablation and there were many proximal lymph nodes measuring less than 1 cm that were left in place, but the lymph nodes greater than 1 cm were resected. The appendix was surgically absent. The large bowel was free of disease. The anterior abdominal wall contained several cystic nodules which were removed. The base of the umbilicus also contained a 2-cm cystic nodule. The right periaortic lymph node measured about 3 cm and was resected. The rest of the lymph nodes were either normal or felt to be less than 0.5 cm and were not resected. At the end of the procedure, optimal tumor debulking was performed to less than 1 cm of disease, with a majority of it being less than 0.5 cm of disease left in place.

**Figure 2:**
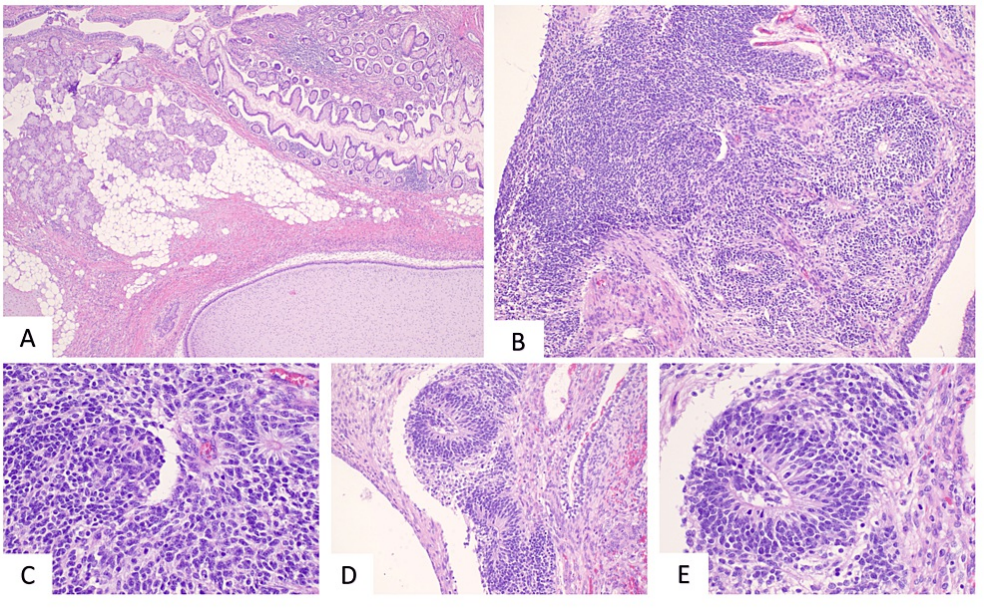
Fallopian tube and ovary - H&E (A) Histologic sections of the right adnexal mass show mature elements including colonic mucosa, ciliated respiratory epithelium, and seromucinous glands, as well as fetal cartilage; (B-E): immature neuroepithelium was also identified in the form of rosettes with areas showing more diffuse growth (occupying >/= 3 low power fields), consistent with a high-grade (grade 3) immature teratoma. H&E: Hematoxylin and eosin

On histopathologic examination of resected tissue, the patient was diagnosed with stage IIIC grade 3 immature teratoma (Figure 2).

She received adjuvant chemotherapy, which consisted of four cycles of bleomycin, etoposide, and cisplatin. Surveillance imaging approximately four months after initial surgery demonstrated a non-avid splenic lesion. CT imaging showed a complex cystic lesion measuring 4.5x5.9cm in the left upper quadrant of the abdomen adjacent to the spleen suggesting residual peritoneal disease. The patient was asymptomatic, and the decision was made to follow up with positron emission tomography (PET) CT imaging given that the lesion could represent chemo-resistant disease versus post-operative changes with peritoneal inclusion cysts.

**Figure 3:**
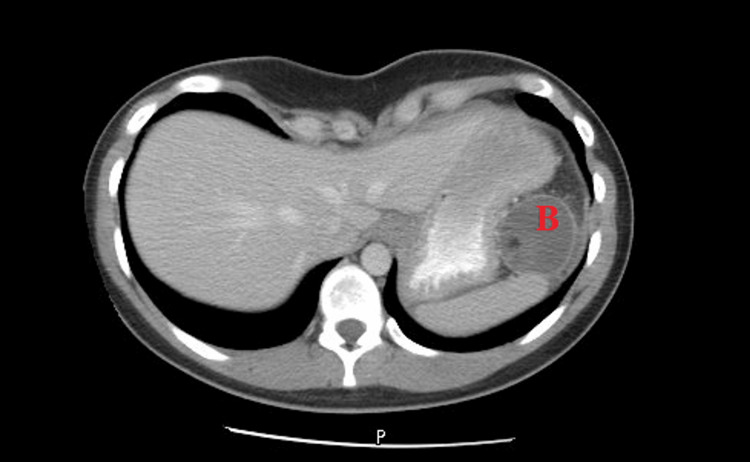
Representative of imaging prior to secondary debulking demonstrating splenic recurrence

Initial imaging showed a decrease in the size of the mass; however, repeat imaging approximately six months from the initial finding of the splenic lesion showed an interval increase in the size of the left upper quadrant cystic lesion to 4.7x4.8cm (Figure 3).

Repeat tumor markers were normal. Given these findings, she underwent a diagnostic laparoscopy with the finding of numerous parietal peritoneal nodules in addition to the splenic lesion. Biopsy of peritoneal nodules returned with final pathology as mature teratoma. Thus, she was taken back to the operating room for robotic splenectomy and partial peritonectomy with a mass noted to be within the splenic hilum.

**Figure 4:**
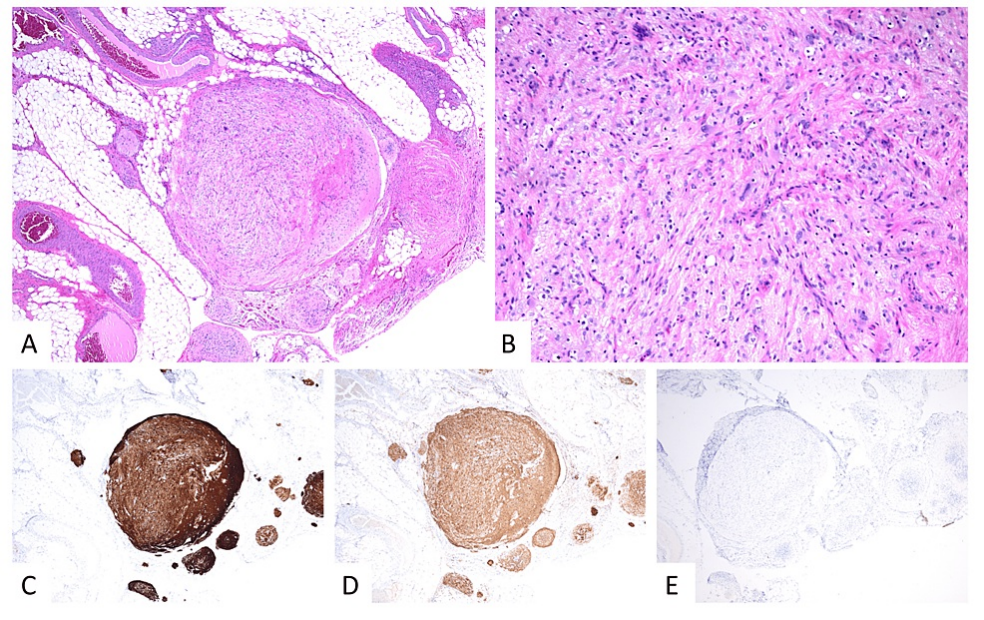
H&E (A-B): Histologic sections of the omentum show nodular studding of predominantly mature glial and focal mature neuronal tissue, also present on the peritoneal surface (not pictured). By immunohistochemistry (IHC), tumor cells are positive for GFAP (C) and S100 (D), consistent with morphologically mature neuroglial origin; IHC for OCT-4 (E) is negative, supporting the absence of an immature neuroepithelial component. Overall findings are consistent with residual mature teratoma presenting as gliomatosis peritonei. GFAP: Glial fibrillary acidic protein, OCT-4: Octamer-binding transcription factor 4, H&E: Hematoxylin and eosin

Spleen pathology showed teratoma consistent with ovarian primary. Final pathology demonstrated mature epithelial, mesenchymal, and neural tissue elements consistent with residual teratoma and no immature elements. The specimens were largely composed of nodules of mature glial tissue and focal areas of mature neuronal tissue. Immunohistochemistry results demonstrated glial fibrillary acidic protein (GFAP) and S100 expression, confirming neural origin tissue. Octamer-binding transcription factor 4 (OCT-4) immunostain was negative which confirmed the absence of immature neural tissue (Figure 4).

The patient recovered from surgery well and remained asymptomatic in the follow-up period of nine months since the last surgical exploration. Most recent CT imaging seven months post-op demonstrated no definite residual mass, and her tumor markers remained normal.

## Discussion

Rare cases of GP with immature teratomas have been reported. Even more rare cases have been reported with mature teratomas [2]. We report a case of rapid progression of immature teratoma with splenic metastasis and peritoneal nodules found to be mature teratoma and associated GP. This finding was following debulking surgery for advanced immature teratoma and four cycles of chemotherapy. Rapid progression of disease with the finding of splenic lesions was initially concerning for this patient after initial histopathology of immature teratoma. Ultimately however based on existing literature, the final pathology revealing mature teratoma with GP is overall reassuring [5]. This case of rapidly progressing teratoma and GP after chemotherapy administration for immature ovarian teratoma is a very rare finding [6]. Prior reports have noted similar findings which can be mistaken for recurrent or progressive disease if not properly diagnosed [6].

General management for GP is surgical resection, however, long-term follow-up and surveillance remain unclear [7]. Some cases described have indicated that patients with extensive peritoneal disease or incomplete resection can be managed with close surveillance due to potential long-term quiescence of GP [5]. Other cases have shown, however, that it is possible to have malignant transformation of mature glial implants years later [3]. The grade and stage of the primary tumor should be used to determine the usage of adjuvant chemotherapy regardless of the presence of GP [8]. Chemotherapy for low-grade GP lesions has not had any evidence-based role to date [8]. Therefore, management and treatment may vary from patient to patient.

## Conclusions

There are no set guidelines for the management and surveillance of teratoma associated with GP. In this case, the patient had a recurrence of immature teratoma ultimately found to be GP with a splenic lesion and peritoneal nodules. This was successfully treated with secondary debulking surgery done via a minimally invasive technique. Recognition of rapidly growing teratoma with new lesions as potential GP is imperative to prevent misdiagnosis as recurrence or progression of disease given the overall reassuring outcomes of GP. Consideration, therefore, should be taken for surgical management for treatment in these patients with full resection, if possible, based on the clinical and radiologic findings as well as surgical feasibility.
